# Subclinical Posttraumatic Stress Disorder Symptoms: Relationships with Blood Pressure, Hostility, and Sleep

**DOI:** 10.1155/2016/4720941

**Published:** 2016-06-15

**Authors:** James A. McCubbin, Heidi M. Zinzow, Melissa A. Hibdon, Aaron W. Nathan, Anastasia V. Morrison, Gregg W. Hayden, Caitlyn Lindberg, Fred S. Switzer

**Affiliations:** ^1^Clemson University, Clemson, SC 29634, USA; ^2^University of South Carolina School of Medicine, Greenville, SC 29605, USA; ^3^University of South Carolina School of Medicine, Columbia, SC 29209, USA

## Abstract

The purpose of this study was to examine the relationships among subclinical PTSD symptoms, blood pressure, and several variables linked to both frank PTSD and the basic psychobiological adaptation to stress. The authors recruited a sample of 91 healthy, young men and women between 18 and 35 years. We examined links among subclinical posttraumatic stress disorder symptoms, blood pressure, sleep quality, and hostility. Posttraumatic stress disorder symptoms were associated with poorer sleep quality and higher hostility scores in both women and men. In men, PTSD symptoms were also associated with elevated resting diastolic blood pressure, and sex was an important moderator of that relationship. Moreover, sleep quality and hostility are substantive mediators of the relationship between diastolic blood pressure and PTSD. Behavioral interventions designed to increase sleep quality and restructure hostile attitudes could potentially serve as preventive interventions for PTSD and the underlying cardiovascular comorbidities in young adults.

## 1. Introduction

The clinical symptomology of posttraumatic stress disorder (PTSD) and the psychobiology of stress and arousal are intimately related. For example, both PTSD and psychological stress are associated with alterations in autonomic, circulatory, and behavioral mechanisms. Patients diagnosed with PTSD often exhibit hyperarousal, blood pressure dysregulation, sleep disruption, and elevated levels of hostility [1–6]. These symptoms are not uncommon during psychological stress in persons without a history of PTSD [7–10], but the precise relationship between the normal, psychobiological adaptations to stress and the expression of PTSD psycho- and physiopathology is not well characterized, especially in young adults. The purpose of this study was to examine, in young men and women, the relationships among subclinical PTSD symptoms and several variables linked to both frank PTSD and the basic psychobiological adaptation to stress, including blood pressure, sleep quality, and hostility. If factors associated with chronic arousal and adaptation to stress are associated with subclinical PTSD-like symptoms, then it may be possible to better understand precursors and potential new preventive and treatment strategies for persons with frank PTSD and its underlying cardiovascular comorbidities in healthy, young adults.

*The Psychobiology of Stress.* Adaptation to acute and chronic psychological stress is accompanied by alterations in the sympathoadrenomedullary and the hypothalamic pituitary adrenocortical systems, with the consequent neuroendocrine cascade affecting blood pressure and a variety of other critical bodily functions. Several factors can influence the magnitude of these changes, including genetic, behavioral, and situational factors. For example, a meta-analysis by Wu et al. [11] found pooled heritability estimates for circulatory stress reactivity ranging from 0.21 to 0.55. Behavioral factors such as hostility have been shown to increase blood pressure during stress [12]. Situational factors influencing stress responsivity include demand, control, and predictability [13]. Moreover, lifestyle and activities also have a significant impact on responses to stress, including aerobic fitness [14] and, importantly, sleep deprivation [15].

*The Psychobiology of PTSD.* PTSD is one potential outcome of extreme stress exposure characterized by autonomic dysregulation, increased cardiovascular reactivity, hyperarousal, sleep disturbances, and hostility. According to DSM-5 criteria, PTSD consists of four clusters of symptoms: (1) intrusion (e.g., intrusive memories); (2) avoidance; (3) negative alterations in cognitions and mood; and (4) alterations in arousal and reactivity [1]. In particular, the arousal and reactivity symptoms may consist of irritable, aggressive, or hostile behavior, hypervigilance, exaggerated startle response, blood pressure dysregulation, and sleep disturbance. Persons with PTSD show a number of psychobiological adaptations that overlap and perhaps interact with the response to nontraumatic acute and chronic stress. Exposure to adverse experiences, including abuse in childhood and adult trauma, can alter neuroendocrine stress reactivity [16]. Moreover, studies of combat veterans with PTSD show alterations in opioidergic mechanisms influencing pain sensitivity as well as blood pressure reactivity [17, 18]. Common risk factors for PTSD are prior trauma, acute stress symptoms, and autonomic hyperarousal, among others [19].

PTSD is associated with catecholamine, serotonin, and hypothalamic pituitary axis (HPA) dysregulation, which contributes to increased sympathetic and circulatory reactivity in response to stressful demands [18, 20, 21]. Researchers hypothesize that PTSD involves chronic overactivation of the sympathetic nervous system [22]. It is therefore not surprising that individuals with PTSD exhibit higher baseline heart rate, blood pressure, and higher incidence of hypertension in comparison to individuals without PTSD [3, 23, 24].

Although hyperarousal and stress reactivity likely represents a mechanistic link between PTSD and blood pressure dysregulation, precise etiologic mechanisms underlying this relationship have not been pointedly examined. Several constructs have been investigated independently as risk factors for hypertension and/or blood pressure dysregulation, including stress reactivity, hostility, and sleep disturbance. Hostility, for example, has been linked to blood pressure control mechanisms [24], risk for coronary heart disease [25], and PTSD [5]. In addition, Beckham and coworkers [24] found that hostile beliefs in women with PTSD were associated with increases in ambulatory blood pressure.

Thus, blood pressure dysregulation, hostility, and sleep disturbances are associated with frank PTSD, as well as responses to stress in persons without a PTSD history. Even though PTSD symptom clusters are generally employed to classify individuals who have been exposed to traumatic events, current research has not fully examined the relationships among those factors in persons without a history of PTSD. Better understanding of the normal relationships among subclinical PTSD symptoms, blood pressure, sleep quality, and hostility in young healthy populations may provide insight into the psycho- and physiopathology underlying adaptations to traumatic stress.

## 2. Methods

### 2.1. Sample and Procedure

Participants between 18 and 35 years of age (average ± SE = 21.4 ± 0.45) provided resting blood pressure levels in a laboratory setting and then completed a series of self-report questionnaires. Participants included a sample of 91 young men (*n* = 42) and women (*n* = 49) recruited from the local geographical area. The final sample was 73.1% white, 9.7% black, 9.7% Asian. Exclusion criteria were history of significant cardiovascular, endocrine, or psychiatric disorder including PTSD or current use of cardiovascular or psychoactive medications.

All procedures were approved by the Institutional Review Board for protection of human research participants, and written informed consent was obtained from all individual participants included in the study. Then, participants were assessed for resting systolic blood pressure (SBP) and diastolic blood pressure (DBP) both sphygmomanometrically and with a calibrated GE Dinamap Pro100V2 automated oscillometric device (GE Medical Systems Information Technologies, Inc., Milwaukee, WI). Dinamap performance was verified on a regular basis for zero offset, integral offset, and gain using a mercury manometer. All resting blood pressure measurements used American Heart Association guidelines for blood pressure determination [26]. Resting blood pressure was determined after a 5 min rest while sitting upright in a comfortable arm chair. For analysis of resting blood pressure, five automated readings of blood pressure and heart rate (HR) were taken at 2 min intervals. The last three automated readings were averaged for analysis to allow stabilization and minimize initial responses to the blood pressure measurement procedure. Participants then completed the PTSD Checklist [27], Pittsburgh Sleep Quality Index (PSQI) [28], and the Cook-Medley Hostility Scale [29].

### 2.2. Measures

*PTSD Checklist.* The civilian version of the PTSD Checklist is a 17-item rating scale incorporating self-report of distress over the past 30 days based on accepted PTSD diagnostic criteria [30]. This scale has been shown to be psychometrically sound for internal consistency, test-retest reliability, discriminant validity, and convergent validity [30, 31]. It has been validated in multiple populations, including motor vehicle accident victims, primary care patients, and community members [32, 33].

*Pittsburgh Sleep Quality Index.* The PSQI is one of the most widely used instruments to measure sleep quality, insomnia, and daytime sleepiness. The PSQI has been systematically evaluated for sensitivity and specificity for sleep disorder screening, is highly related to sleep diary data, and has been assessed in both population-based and clinical studies [28]. PSQI scores are sensitive to blood pressure and catecholamine levels in hypertensive patients [34].

*Cook-Medley Hostility Scale.* The Cook-Medley Hostility Scale is a widely used self-report assessment including suspiciousness, resentment, and cynical mistrust, rather than overt aggressive behaviors [29, 35]. Persons with high levels of hostility show increased blood pressure responses to stress and increased anger [12, 35]. The Cook-Medley Hostility Scale has been shown to have good convergent and discriminant validity and has been linked to coronary heart disease and all-cause mortality [29].

### 2.3. Statistical Analysis

Results were analyzed using SPSS for descriptive statistics and linear regression analyses. These data were analyzed for the total group and for men and women separately. Regression analyses utilized PTSD symptoms as the dependent variable, with age, sex, blood pressure, hostility, and sleep quality as independent variables. Mediation and moderation were assessed by the moderated causal steps approach [36].

## 3. Results

Table 1 shows descriptive statistics for the total sample and separately for men and women. The present sample of young adults averaged 21.4 ± 0.45 years of age (mean ± SE). Men averaged 21.7 years while women averaged 21.2 years, with no significant sex differences. Average blood pressure in the total study sample was 113.7 ± 1.30 mmHg for systolic and 67.6 ± 0.60 for diastolic blood pressure. The average SBP for men was 121.1 ± 1.50 while women averaged 107.4 ± 1.59 mmHg, and this difference was statistically significant (*p* < .001). There were no significant sex differences for DBP, PSQI scores, or PTSD symptoms. However, the average hostility score was significantly higher (*p* = .006) for men (21.5 ± 1.17) than for women (17.3 ± 0.96).

**Figure 1:**
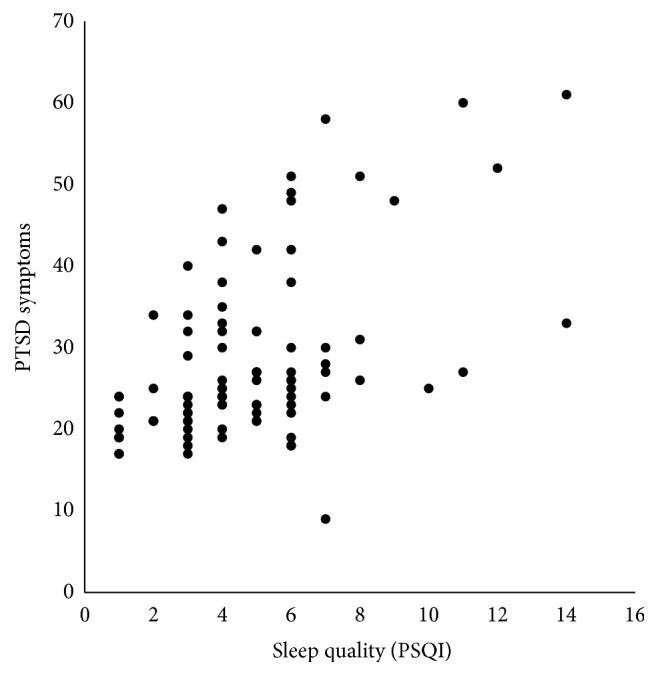
Scatter plot of the relationship between sleep quality as measured by the Pittsburgh Sleep Quality Index (PSQI; higher scores indicate poorer quality of sleep) and PTSD Symptoms as measured by the Posttraumatic Stress Disorder Checklist (higher scores indicate more symptoms reported) in men and women combined (*r*(88) = 0.517, *p* < .001).

**Figure 2:**
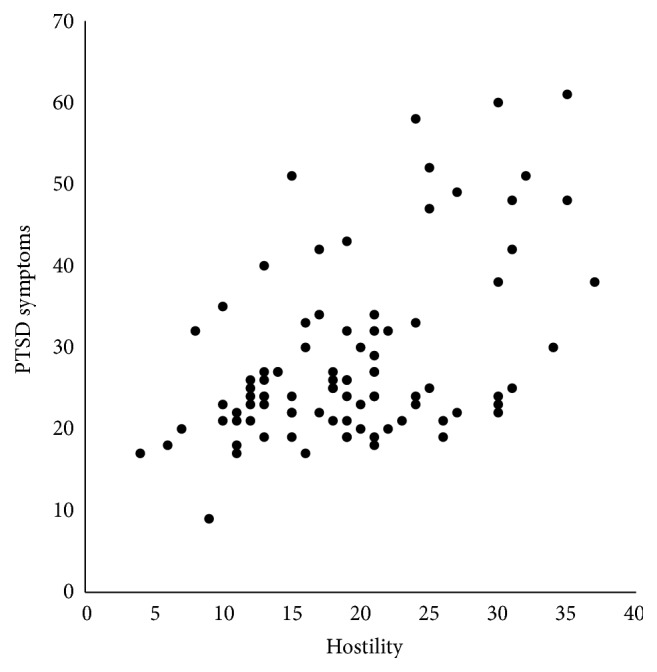
Scatter plot of the relationship between hostility as measured by the Cook-Medley Hostility Scale (higher scores indicate higher hostility) and PTSD Symptoms as measured by the Posttraumatic Stress Disorder Checklist (higher scores indicate more symptoms reported) in men and women combined (*r*(86) = 0.492, *p* < .001).

Zero-order correlations (see Table 2) among the study variables showed that PTSD symptoms were strongly associated with poorer sleep quality (*r*(88) = .517, *p* < .001) and higher hostility scores (*r*(86) = .492, *p* < .001) in the total sample. These relationships are illustrated in Figures 1 and 2. Age was also positively associated with diastolic blood pressure in the full sample (*r*(87) = .221, *p* = .038). In young women (see Table 3), PTSD symptoms were associated with high hostility scores (*r*(46) = .362, *p* = .011) and poor sleep quality (*r*(47) = .303, *p* = .034). In young men, PTSD symptoms were associated with poor sleep quality (*r*(39) = .691, *p* < .001) and higher hostility scores (*r*(38) = .597, *p* < .001) and were also associated with higher resting diastolic blood pressure (*r*(39) = .402, *p* = .009).

Multiple regression analyses were conducted to examine unique contributions of study variables to PTSD symptoms. The models included age, resting diastolic blood pressure, hostility, and sleep quality as independent variables (see Table 4). Three separate sets of regression analyses were conducted, using the total sample and men and women separately. Results indicated that both hostility and sleep quality were independently associated with PTSD symptoms in the total sample (*p* < .001), and in men (*p* < .01). Hostility remained a unique correlate of PTSD symptoms in women (*p* = .025), while the effect of sleep quality revealed a strong trend (*p* = .052). In men, both sleep quality and hostility remained significant (*p* = .01 and *p* < .001, resp.), whereas diastolic blood pressure was no longer significant when both variables were included in the model (*p* = .564).

Systematic analysis of mediation and moderation [36] indicated that sex is an important moderator of the relation between diastolic blood pressure and PTSD. Moreover, sleep quality and hostility are substantive mediators of the relationship between diastolic blood pressure and PTSD symptoms. Results showed a much stronger relation between sleep and PTSD for men (*R* ^2^ = .48) and a milder relation for women (*R* ^2^ = .09).

## 4. Discussion

The present study was designed to examine the relationships among subclinical PTSD symptoms and several variables linked to both frank PTSD and the basic psychobiological adaptation to stress in young adults. Subclinical PTSD symptoms have been studied in survivors of intimate partner violence [37], Vietnam veterans [38], and young adults [39], among others, but these studies have not focused on factors directly related to the psychobiology of stress and arousal. However, preclinical studies of the relationship between PTSD-associated symptoms, blood pressure, sleep quality, and hostility are important for several reasons. First, relatively little work has been done to characterize these relationships in the normal population, a scientific step necessary to fully understand the nature of biobehavioral processes involved in the etiology of PTSD physio- and psychopathology upon exposure to traumatic stress. Second, a better understanding of the basic effects of common stress-associated biobehavioral variables may provide some insight into preclinical factors that may lead to vulnerability or increased risk of PTSD development. And third, a better understanding of stress mechanisms that interact with PTSD symptomology may provide insight into new strategies to treat PTSD and its underlying cardiovascular comorbidities. Even more importantly these findings may potentially inform new strategies to develop resilience in, for example, combat troops prior to deployments with high potential for exposure to combat-related trauma.

There are several remarkable results from the current study. Diastolic blood pressure is a substantive predictor of PTSD symptoms in men, but not in women. The relationship between diastolic blood pressure and PTSD in men is strongly mediated by sleep quality and to a lesser extent by hostility. In women, sleep quality and hostility predict PTSD (with hostility being a slightly stronger predictor), but BP is not related to either of these, nor to PTSD symptom expression. These findings are particularly interesting since there is a significant literature linking sleep quality and hostility with (1) psychophysiological responses to acute and chronic stress and (2) risk for cardiovascular diseases often found comorbid with PTSD.

### 4.1. Sleep Quality

Our finding that poor sleep quality was associated with higher blood pressure and PTSD symptoms is consistent with prior research examining autonomic dysregulation underlying both sleep dysfunction and physiological responses to stress. Several investigations from our laboratory and others have found that sleep quality is associated with autonomic dysregulation and elevated blood pressure. For example, epidemiologic studies have documented an association between blood pressure elevations and both shorter sleep duration and lower sleep maintenance [40–42]. Laboratory evidence suggests that increased sympathetic nervous system activity is a mechanism underlying the association between poor sleep quality and blood pressure elevations [43–45]. Studies of sleep deprivation from our laboratory [15] suggest that acute sleep deprivation can contribute to blood pressure elevations in persons at risk for hypertension. Other work indicates that the relationship between sleep deprivation and blood pressure is associated with increased autonomic arousal. For example, in a microneurographic study, Ogawa and associates [44] indicate that 24 hours of total sleep deprivation elevates blood pressure through resetting of arterial baroreflexes. Moreover, persons with insomnia show reduced heart period and heart period variability, with increased low frequency and decreased high frequency power spectra, suggesting that sleep deprivation may alter parasympathetic tone on the heart [45]. Therefore, chronic sleep deprivation, whether from short sleep duration or circadian desynchronization, appears to alter the balance between the sympathetic and parasympathetic nervous systems.

### 4.2. Hostility

Our finding that hostility was associated with sleep quality, blood pressure, and PTSD symptoms adds to the literature on potential psychosocial mechanisms accounting for the relation between stress responses, autonomic dysregulation, and symptom expression. Meta-analysis has documented a large effect size for the association between hostility and PTSD, which increased over time since the traumatic event [5]. Individuals high on trait hostility may be particularly vulnerable to developing PTSD symptoms and sympathetic nervous system overactivation [46]. The hostility literature also indicates an intimate relationship between hostility and both circulatory disease risk [25] and blood pressure reactivity [24]. Thus, our findings that hostility could account for the relationship between blood pressure and PTSD symptoms in men are consistent with existing literature.

### 4.3. Autonomic Nervous System Arousal

The increased arousal resulting from hostility and poor sleep quality may operate in synergy with the physiological response to traumatic stress in production of PTSD symptoms. PTSD symptoms are associated with both hostility and sleep disturbance, which, based on our current findings, could account for the autonomic dysregulation and increased risk for hypertension observed in individuals with PTSD. PTSD involves chronic overactivation of the sympathetic nervous system [22], in addition to concomitant alterations in parasympathetic control of the heart. It is therefore not surprising that individuals with PTSD exhibit higher baseline heart rate and blood pressure values in comparison to individuals without PTSD [2–4]. The finding that hostility and sleep disturbance were related to PTSD symptoms in the overall sample, and that blood pressure was related to both sleep disturbance and PTSD symptoms in men, supports the theory that PTSD involves sympathetic nervous system overactivation. Our data suggest that, at least in men, the relationship between resting blood pressure and PTSD symptoms is secondary to the effect of poor sleep quality and hostility. Nevertheless, the precise causal pathways cannot be definitively determined from the current methodology. Additional future work such as randomized trials of both sleep-enhancing interventions and cognitive restructuring of hostile attitudes is recommended to better understand the causal nature of the relationships among sleep quality, hostility, blood pressure control, and PTSD symptom expression in populations who may be at risk for subsequent exposure to traumatic stressors.

### 4.4. Potential Efficacy of Stress Management

Evidence for the potential efficacy of stress management comes from research showing that cognitive behavioral stress management (CBSM) training, especially in small groups, reduces hostility and related psychosocial and physiological characteristics. For example, Bishop and coworkers found that psychosocial skills training reduced anger as well as depression, social isolation, and BP at rest and during anger recall stress in patients following coronary artery bypass graft [47]. Moreover, two independent randomized, controlled trials showed that cognitive behavioral therapy reduced mortality in patients with coronary heart disease [48, 49]. An observational trial in multiple corporate worksites showed that such training produced reductions in hostility that were maintained six months following the completion of training [50]. Additional future work such as randomized trials of both sleep-enhancing interventions and cognitive restructuring of hostile attitudes is recommended to better understand the causal nature of the relationships among sleep quality, hostility, blood pressure control, and PTSD symptom expression in men and women without a reported history of frank PTSD.

### 4.5. Limitations

This study does not directly address causal connections among study variables. It is likely that the relationships among the study variables are bidirectional in nature, and thus causal influences may be difficult to fully characterize. Longitudinal studies are needed to investigate the nature of these relationships over time. Another limitation is that the study sample was not sufficient to examine the potential effects of race and/or ethnicity. While the current subclinical sample is valuable for examination of precursors to PTSD symptom expression, the results may not generalize to other age groups and/or all racial, ethnic, and socioeconomic populations. Furthermore, it is possible that individuals meeting clinical criteria for PTSD are subject to additional autonomic irregularities and other psychosocial risk factors compared with the subclinical population included in this study. On the other hand, effect sizes in this study may represent underestimates of the effects that may be observed in clinical populations. Finally, caution should be taken in etiologic interpretation of our findings, as it can be difficult to disentangle these constructs in causally meaningful ways without further and more extensive study.

## 5. Conclusions

In summary, these findings suggest that poor sleep quality and hostility are associated with subclinical PTSD symptoms in healthy young men and women without a known history of PTSD. In young men, elevated blood pressure was associated with more PTSD symptoms, but this effect appeared to be accounted for, at least in part, by both sleep quality and hostility. Thus sleep quality and hostility may underlie some of the hyperarousal and autonomic nervous system disturbance in the expression of clinical PTSD symptoms, at least in men.

The fact that both hostility and sleep disturbance were associated with PTSD symptoms independent of blood pressure suggests that they may represent underlying psychobiological mechanisms by which trauma relates to hyperarousal. Sleep quality and hostility accounted for the relation between diastolic blood pressure and PTSD symptoms in men but not women, so it is possible that different psychobiological processes underlie stress responses and autonomic dysregulation in men versus women. For example, researchers have described an oxytocin-associated “tend and befriend” approach to stress among women, in addition to the traditional fight-or-flight response that is more prevalent in men [51]. Future research is necessary to further explore gender differences in responses to chronic stress that may contribute to PTSD risk. Finally, these data raise the possibility that behavioral interventions to enhance sleep quality and restructure hostile attitudes could potentially serve as effective interventions for PTSD and its underlying cardiovascular comorbidities. If further work verifies these causal mechanisms, then our data suggest that interventions directed at sleep quality and hostile attitudes may relieve symptoms in persons with PTSD and may possibly provide new strategies for building resilience in persons who are likely to be exposed to traumatic stress in the future.

## Figures and Tables

**Table 1:** Descriptive statistics for the total sample and for men and women separately.

	*N*	Minimum	Maximum	Mean		Std. deviation
	Statistic	Statistic	Statistic	Statistic	SE	Statistic
*Total sample*						
Age	89	18	52	21.40	.454	4.285
Sleep quality	91	1	14	4.82	.283	2.702
DBP	91	53.7	84.0	67.564	.6004	5.7272
SBP	91	86.3	141.7	113.689	1.2971	12.3738
PTSD symptoms	92	9	61	28.32	1.098	10.535
Hostility	89	4	37	19.24	.775	7.307

*Men*						
Age	42	18	33	21.69	.490	3.174
Sleep quality	42	1	14	4.86	.452	2.927
DBP	42	57.0	84.0	67.876	.9188	5.9544
SBP	42	99.3	141.7	121.090	1.4448	9.3636
PTSD symptoms	41	17	61	29.56	1.876	12.011
Hostility	41	6	37	21.51	1.170	7.490

*Women*						
Age	47	18	52	21.15	.744	5.099
Sleep quality	49	1	14	4.80	.361	2.525
DBP	49	53.7	80.3	67.296	.7962	5.5731
SBP	49	86.3	139.3	107.345	1.5855	11.0983
PTSD symptoms	49	9	58	27.49	1.332	9.325
Hostility	48	4	35	17.29	.956	6.620

**Table 2:** Zero-order correlations among study variables in women and men combined.

		Age	Sleep quality	DBP	SBP	PTSD symptoms	Hostility
Sex (men = 0, women = 1)	Pearson correlation	−.063	−.011	−.051	−.557^*∗∗*^	−.098	−.290^*∗∗*^
	Sig. (2-tailed)	.555	.915	.633	.000	.360	.006
	*N*	89	91	91	91	90	89

Age	Pearson correlation		−.070	.221^*∗*^	.087	−.029	.031
	Sig. (2-tailed)		.516	.038	.417	.788	.776
	*N*		89	89	89	88	87

Sleep quality	Pearson correlation			.251^*∗*^	.116	.517^*∗∗*^	.270^*∗*^
	Sig. (2-tailed)			.017	.272	.000	.010
	*N*			91	91	90	89

DBP	Pearson correlation				.515^*∗∗*^	.202	.173
	Sig. (2-tailed)				.000	.056	.105
	*N*				91	90	89

SBP	Pearson correlation					.116	.163
	Sig. (2-tailed)					.276	.126
	*N*					90	89

PTSD symptoms	Pearson correlation						.492^*∗∗*^
	Sig. (2-tailed)						.000
	*N*						88

^*∗∗*^Correlation is significant at the 0.01 level (2-tailed).

^*∗*^Correlation is significant at the 0.05 level (2-tailed).

**Table 3:** Zero-order correlations for women (above diagonal) and men (below diagonal).

		Age	Sleep quality	DBP	SBP	PTSD symptoms	Hostility
Age	Pearson correlation		−.076	.176	.085	−.150	−.007
	Sig. (2-tailed)	—	.614	.236	.571	.313	.966
	*N*		47	47	47	47	46

Sleep quality	Pearson correlation	−.076		.135	−.029	.303^*∗*^	.101
	Sig. (2-tailed)	.633	—	.354	.845	.034	.495
	*N*	42		49	49	49	48

DBP	Pearson correlation	.308^*∗*^	.360^*∗*^		.683^*∗∗*^	−.036	.047
	Sig. (2-tailed)	.047	.019	—	.000	.805	.752
	*N*	42	42		49	49	48

SBP	Pearson correlation	.019	.329^*∗*^	.471^*∗∗*^		−.077	−.048
	Sig. (2-tailed)	.904	.033	.002	—	.601	.745
	*N*	42	42	42		49	48

PTSD symptoms	Pearson correlation	.126	.691^*∗∗*^	.402^*∗∗*^	.244		.362^*∗*^
	Sig. (2-tailed)	.434	.000	.009	.124	—	.011
	*N*	41	41	41	41		48

Hostility	Pearson correlation	.048	.442^*∗∗*^	.285	.068	.597^*∗∗*^	
	Sig. (2-tailed)	.763	.004	.071	.671	.000	—
	*N*	41	41	41	41	40	

^*∗*^Correlation is significant at the 0.05 level (2-tailed).

^*∗∗*^Correlation is significant at the 0.01 level (2-tailed).

**Table 4:** Multiple regression predicting PTSD symptoms from age, sex, diastolic blood pressure, hostility, and sleep quality.

Model	Unstandardized coefficients		Standardized coefficients	*t*	Sig.
	B	Std. error	Beta		
*Total sample*					
(Constant)	6.745	11.318		.596	.553
Age	−.030	.222	−.012	−.137	.892
Sex	.613	1.949	.028	.315	.754
DBP	.049	.174	.026	.284	.777
Hostility	.546	.141	.367	3.865	.000
Sleep quality	1.721	.385	.421	4.474	.000

*Men*					
(Constant)	−13.337	15.127		−.882	.384
Age	.508	.425	.135	1.196	.240
DBP	.142	.244	.071	.582	.564
Hostility	.539	.197	.328	2.739	.010
Sleep quality	2.194	.514	.533	4.267	.000

*Women*					
(Constant)	27.398	16.191		1.692	.098
Age	−.208	.262	−.112	−.794	.432
DBP	−.132	.244	−.078	−.542	.591
Hostility	.465	.200	.323	2.329	.025
Sleep quality	1.151	.574	.285	2.006	.052
